# In vivo functional analysis of non-conserved human lncRNAs associated with cardiometabolic traits

**DOI:** 10.1038/s41467-019-13688-z

**Published:** 2020-01-02

**Authors:** Xiangbo Ruan, Ping Li, Yi Chen, Yu Shi, Mehdi Pirooznia, Fayaz Seifuddin, Hiroshi Suemizu, Yasuyuki Ohnishi, Nao Yoneda, Megumi Nishiwaki, James Shepherdson, Abhilash Suresh, Komudi Singh, Yonghe Ma, Cheng-fei Jiang, Haiming Cao

**Affiliations:** 1 Cardiovascular Branch, National Heart, Lung and Blood Institute, National Institutes of Health, Bethesda, MD 20892 USA; 2 Bioinformatics and Computational Biology Core, National Heart Lung and Blood Institute, National Institutes of Health, Bethesda, MD 20892 USA; 3 Laboratory Animal Research Department, Biomedical Research Laboratory, Central Institute for Experimental Animals, 3-25-12 Tonomachi, Kawasaki-ku, Kawasaki 210-0821 Japan; 4 Technical Service Department, CLEA Japan, Inc, 4839-23 Kitayama, Fujinomiya, Shizuoka 418-0122 Japan; 5 Present Address: Division of Biology and Biomedical Sciences, Washington University School of Medicine, St. Louis, Missouri USA

**Keywords:** Genetics, Molecular biology, Long non-coding RNAs, Diseases

## Abstract

Unlike protein-coding genes, the majority of human long non-coding RNAs (lncRNAs) are considered non-conserved. Although lncRNAs have been shown to function in diverse pathophysiological processes in mice, it remains largely unknown whether human lncRNAs have such in vivo functions. Here, we describe an integrated pipeline to define the in vivo function of non-conserved human lncRNAs. We first identify lncRNAs with high function potential using multiple indicators derived from human genetic data related to cardiometabolic traits, then define lncRNA’s function and specific target genes by integrating its correlated biological pathways in humans and co-regulated genes in a humanized mouse model. Finally, we demonstrate that the in vivo function of human-specific lncRNAs can be successfully examined in the humanized mouse model, and experimentally validate the predicted function of an obesity-associated lncRNA, LINC01018, in regulating the expression of genes in fatty acid oxidation in humanized livers through its interaction with RNA-binding protein HuR.

## Abstract (web summary)

Majority of human long non-coding RNAs (lncRNAs) are not conserved in mouse. Here the authors identify metabolic trait-associated lncRNA genes and show a functional role of a non-conserved human lncRNA, LINC01018, in lipid metabolism using a humanized mouse model.

## Introduction

The past decade has seen unprecedented progress in our understanding of the human genome, unraveling the widespread expression of long non-coding RNAs (lncRNA) that have emerged as key regulators of gene expression^1,2^. LncRNAs are transcripts that are over 200 nucleotides long and lack any predicted coding potential. Presently, lncRNAs have been identified in all model organisms and between 30,000 and 60,000 human lncRNAs have been reported in recent lncRNA annotations^3,4^. Compared to mRNAs, lncRNAs exhibit stronger tissue-specificity and often function in a tissue-specific manner^5^. Cellular experiments have shown that lncRNAs are involved in a wide spectrum of biological processes ranging from cell proliferation, apoptosis and nutrient sensing to cell differentiation^1,6^. A prevailing mechanism through which lncRNAs exert their function is by interacting with transcription factors or chromatin-modifying complexes to modulate gene transcription^2^. Moreover, a growing number of lncRNAs have been reported to play an important role in a wide range of pathophysiological processes at organismal level in research animals^7–12^, suggesting that defining the function of human lncRNAs could provide substantial insights into human biology and physiology.

However, unlike protein-coding genes, which are largely conserved in mammals, vast majority of human lncRNAs are non-conserved, and many of them are probably lineage-specific^13–16^. Although the low conservation of genomic sequences often suggests a limited or lack of function, emerging evidence supports that some non-conserved human lncRNAs are clearly functional, at least in cultured cells^17,18^. Moreover, recent examples of structurally but not sequentially conserved lncRNAs^19,20^ suggest that we might not yet understand the mode of lncRNA conservation. Presently, the primary tool to evaluate gene conservation is the BLAST heuristic, which relies on contiguous stretches of alignable sequence for conservation detection. LncRNAs, however, could potentially be conserved at many different levels^21^ in additional to their sequence such as secondary or high-order structure, short motifs for protein binding, position for lncRNAs whose transcription but not transcript is functional^22^, and none of these can be effectively identified by BLAST. Thus we currently do not know how many lncRNAs are functionally conserved and cannot use existing comparative genomic tools to identify the specific functional role of human lncRNAs. A validated system is clearly needed to enrich potentially functional human lncRNAs and to infer their role in specific biological processes for further experimental characterization. Furthermore, a suitable experimental system is required to directly define the physiological function of non-conserved human lncRNAs. Cell culture is often incapable of modeling complex physiological environments, and an in vivo system is needed to definitively define a gene’s function. So the physiological function of human protein-coding genes is often studied in animals particularly mice in which over 90% of human protein-coding genes are conserved. Unfortunately, an in vivo experimental system that is suitable to study non-conserved human lncRNAs is currently unavailable.

In this study, we establish a practical pipeline for studying the physiological function of non-conserved human lncRNAs. We first identify a list of human lncRNAs that are specifically regulated by genome-wide association study (GWAS) loci of metabolic diseases in the liver. We then enhance the selection of potentially functional lncRNAs using information from epigenetic markers and 3-D chromatin interactions, liver enrichment, co-expression functional prediction, and in vivo regulation by metabolic milieu. We further confirm that the regulation and function of non-conserved human lncRNAs can be defined in a liver-specific humanized mouse model. Encouragingly, we successfully validate our pipeline by identifying the function of a non-conserved human lncRNA that is regulated by a GWAS locus associated with increased obesity risk.

## Results

### Identification of cardiometabolic traits related lnc-eGenes

Integration of GWAS and eQTL signals has led to the identification of functional genes for disease susceptibility^23^, but these efforts have been traditionally focused on protein-coding genes. Here we leverage this critical data source to identify functional lncRNAs that are regulated by GWAS-eQTL colocalized loci.

**Fig. 1:**
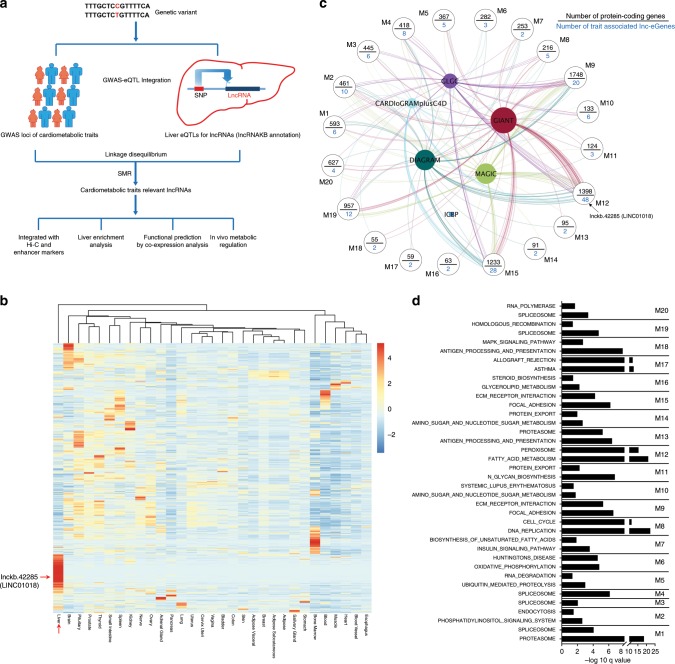
Identification and functional prediction of human liver lnc-eGenes. **a** Flowchart depicting a pipeline for identifying potentially functional human lncRNAs by integration of GWAS and eQTL data and by further bioinformatic and experimental analyses. **b** Heatmap of tissue expression Z score for the cardiometabolic trait-associated lnc-eGenes. LINC01018 and liver tissue were pointed out by red arrows. **c** Co-expression module analysis of cardiometabolic trait-associated lnc-eGenes. The modules with at least one significant KEGG pathway and including 2 or more trait associated lnc-eGenes are shown. **d** KEGG pathway analysis of protein-coding genes in each module; only the top two most significant pathway for each module are displayed.

We are particularly interested in human lncRNAs that regulate metabolism in the liver, an organ that is central to metabolism and also highly relevant to an array of cardiometabolic disorders. Thus, we attempt to systemically map human lncRNAs regulated by the eQTLs in the liver that colocalize with GWAS loci of major cardiometabolic diseases or traits (Fig.1a and Supplementary Data 1). Although the number of reported lncRNAs is enormous, only a fraction of them have been systemically interrogated by eQTL mapping using RNA-seq-based gene expression profiling to identify eQTL-regulated lncRNA genes (lnc-eGene). To expand lncRNA coverage in eQTL calculation, we re-analyzed human liver RNA-seq data from Genotype-Tissue Expression (GTEx) project using the most updated and comprehensive human lncRNA annotation, lncRNA Knowledgebase (lncRNAKB) (10.1101/669994). cis-eQTLs for liver-expressed lncRNAs were then calculated based on the genotyping data of the GTEx consortium (see “Methods”). Due to complicated linkage disequilibrium in the human genome, we applied method of HEIDI (heterogeneity in dependent instruments) implemented in SMR (Summary-data-based Mendelian Randomization) analysis^24^ to determine if our identified eQTLs of lncRNAs are functionally colocalized with the GWAS signals. In total, 726 unique lnc-eGenes whose eQTLs in liver are colocalized with at least one nominally significant GWAS SNPs (single nucleotide polymorphisms) were identified (Supplementary Data 1), suggesting their expression in liver are potentially associated with cardiometabolic traits. Consistent with our current understanding that the majority of human lncRNAs are non-conserved^13^, the average conservation score of these trait-associated lnc-eGenes is only around 0.12 based on multiple alignment of 30 vertebrate species (see “Methods”).

Next, we used several additional analyses to enhance the selection of functional lncRNAs (Fig. 1a). First, to gain further evidence that these traits-associated lnc-eGenes are subjected to direct regulation of their eQTL/GWAS colocalized loci, we integrated H3K4me1 and/or H3K27ac ChIP data and high-throughput chromatin conformation capture (Hi-C) data from the human liver^25^ (see “Methods”). As shown in Supplementary Data 2, for 44% (320 out of 726) lnc-eGenes, spatial chromatin interactions between their gene regions and eQTL/GWAS colocalized loci showing active enhancer marker were observed. This result suggests a substantial fraction of these eQTL/GWAS loci might act as enhancers to regulate the expression of these lnc-eGenes. Second, the enriched expression of lncRNAs in a specific tissue could suggest their unique functions. To determine the liver-enrichment of the identified lnc-eGenes, the raw RNA-seq data across different tissues in GTEx were re-analyzed, and a Z score was calculated to determine the expression enrichment of each lncRNA (Fig. 1b and Supplementary Data 3). Around 16% of the identified lnc-eGenes have a liver Z score more than 2. Moreover, the mean liver expression Z scores of these lnc-eGenes are significant higher than the ones of the random sets of liver-expressed genes (see “Methods”), suggesting these lnc-eGenes exhibit stronger liver-enriched expression and may play a specific role in hepatic metabolism. Third, we use co-expression network analysis of liver transcriptome to infer the potential function of trait-associated lncRNAs. As shown in Fig. 1c, the co-expression module identification tool (CEMiTOOL)^26^ was used to group these trait-associated lncRNAs into different functional modules. KEGG Pathway analysis for the co-expressed protein-coding genes in each module showed these lncRNAs may function in diverse metabolic pathways including fatty acid metabolism, steroid biosynthesis, mRNA processing and amino acid metabolism (Fig. 1d and Supplementary Data 4).

Taken together, we have identified a list of cardiometabolic trait-associated lnc-eGenes which are mostly non-conserved and could potentially function in diverse metabolic pathways in human liver.

### A model to study the in vivo regulation of human liver genes

As most trait-associated human lncRNAs identified above are non-conserved, conventional mouse models are not a suitable tool to study their in vivo regulation and function. To address this challenge, we sought to employ a recently developed humanized TK-NOG mouse model where the liver is repopulated with human hepatocytes^27^. One clear advantage of the humanized mice is that the changes in human hepatic gene expression can be examined under well-controlled experimental conditions, and more importantly, under a uniform genetic background, which is impractical in human populations. Such changes in lncRNA expression levels in response to metabolic stimuli could provide critical clues about their roles in metabolism, facilitating their downstream functional analysis.

**Fig. 2:**
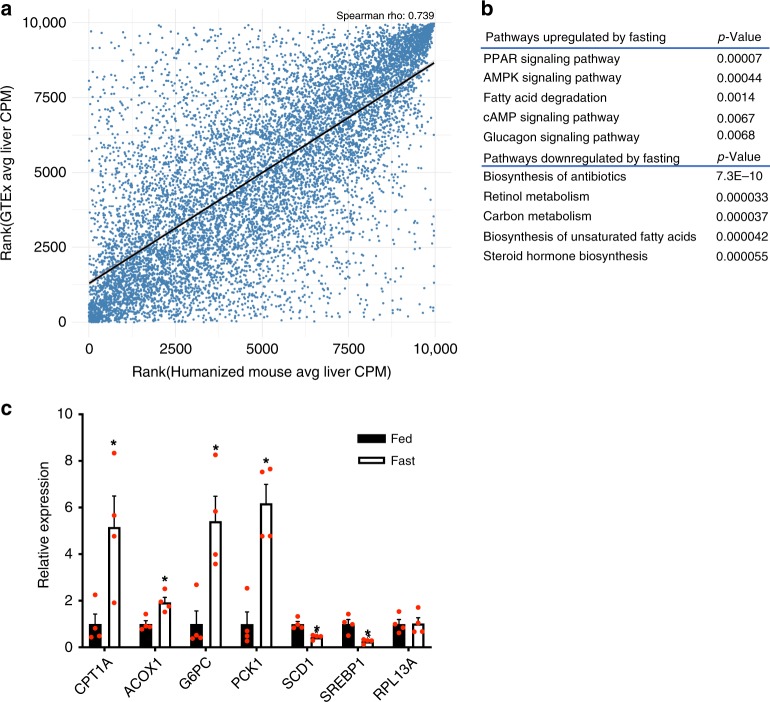
In vivo regulation of human hepatic genes in a humanized mouse model. **a** Spearman correlation analysis of RNA-seq data from human liver tissue and humanized liver. The blue dots represent all human genes expressed in the liver. **b** Representative pathways overrepresented by human protein-coding genes regulated by fasting in humanized liver. **c** Expression levels of representative genes of major metabolic pathway in the livers of humanized mice fed ad libitum (Fed, *n* = 4), subject to a 24-h fast (Fast, *n* = 4). Data in (**c**) represent mean ± SEM, *p < 0.05, two-tailed unpaired Student’s *t*-test. Source data of (**c**) are provided in the Source Data file.

To assess the feasibility of using this humanized mouse to study the physiological function of non-conserved human lncRNAs, we first examined if the human genes in humanized liver maintain a physiological response to metabolic milieus such as fasting. We performed a deep RNA-seq analysis of liver tissues of humanized mice with a 24 h food withdrawal (fasting) or fed ad libitum (fed). In order to analyze the human-specific transcriptome in these chimeric livers, the RNA-seq reads were first mapped against a combined human and mouse genome, and only human-specific reads were used for further analysis (see Methods). We then performed a correlation analysis of gene expression in humanized livers and in human livers and found the expression levels of human protein-coding and non-coding genes are comparable (Fig. 2a, the Spearman’s rho is 0.739), indicating the humanized liver can faithfully reflect human liver biology at least at the transcript level. Since the understanding of hepatic gene expression in response to fasting is more established for protein-coding genes than that for lncRNA genes, we then used fasting-regulated human hepatic protein-coding genes to assess the functionality of the humanized liver. A pathway analysis showed that the upregulated pathways include PPAR signaling, fatty acid degradation, and glucagon signaling pathways whereas the down-regulated pathways include carbon metabolism, biosynthesis of unsaturated fatty acids and steroid hormone biosynthesis pathway, which is fully in line with known hepatic response to fasting (Fig. 2b). The expression changes of several key genes in these pathways were further confirmed by quantitative real-time PCR (qPCR) using human-specific primers (Fig. 2c). These results support that human hepatocytes in the humanized mice maintain their physiological function during fasting. Moreover, the in vivo regulation of trait-associated lncRNAs by metabolic conditions is another layer of useful information to infer their physiological functions. It is noticed that 32 lnc-eGenes are subjected to the regulation by fasting in vivo (Supplementary Data 5), providing a clue that they may function in fasting-related metabolism regulation.

### Defining the in vivo function of a trait-associated lncRNA

**Fig. 3:**
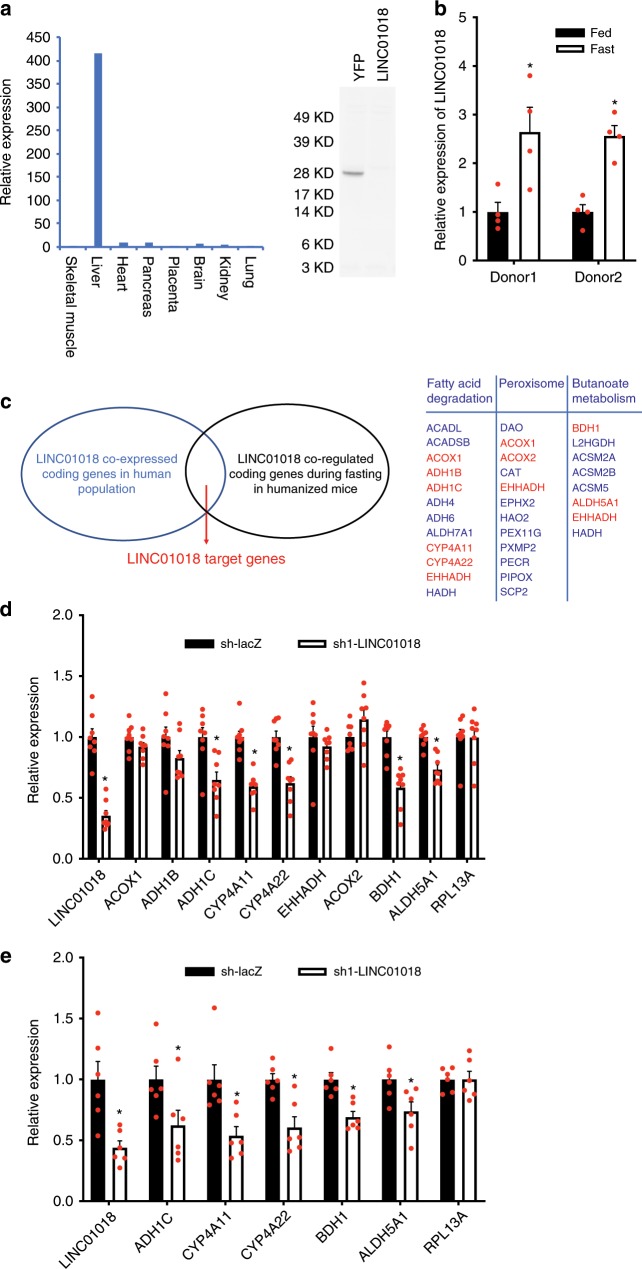
Define the in vivo molecular function of a trait-associated lncRNA. **a** Left: Relative expression levels of LINC01018 in human tissues of skeletal muscle (set as 1), liver, heart, pancreas, placenta, brain, kidney and lung analyzed by quantitative real-time PCR using human cDNA tissue panel from Clontech. Right: in vitro translation analysis using RNAs of YFP and LINC01018. **b** Hepatic expression of LINC01018 in response to fasting in humanized mice (*n* = 4 for each group) generated with human primary hepatocytes from two different donors. **c** Left: an illustration depicting the strategy to identify potential LINC01018 target genes. Right: in top pathways overrepresented by LINC01018 correlated protein-coding genes, the potential LINC01018 target genes are highlighted in red, which are also regulated by fasting in humanized mice together with LINC01018. **d** Expression levels of LINC01018 and its potential target genes in the livers of control and LINC01018 knockdown (KD) humanized mice received shRNA adenoviruses for LacZ (LacZ sh, *n* = 8) and LINC01018 (LINC01018 sh1, *n* = 8) respectively. **e** Gene expression in the livers of control (LacZ sh, *n* = 6) and LINC01018 KD (LINC01018 sh1, *n* = 6) humanized mice using human primary hepatocytes from a second donor after a 24 h food withdrawal. Data in (**b**, **d**, **e**) represent mean ± SEM, *p < 0.05, two-tailed unpaired Student’s *t*-test. Source data of (**a**, **b**, **d**, **e**) are provided in the Source Data file.

After establishing a selected list of human lncRNAs with high functional potential and characterizing a humanized liver model where human hepatic genes exhibit proper responses to nutrient states, we have established an experimental pipeline that is uniquely suitable for interrogating the functional significance of our identified trait-associated lncRNAs. Among the identified lncRNAs, we were particularly interested in lnckb.42285 (LINC01018), a non-conserved intergenic lncRNA on Chromosome 5 (Supplementary Fig. 1a). Multiple lines of evidence from our pipeline suggest that LINC01018 could play a role in metabolism: GWAS-eQTL integrative analysis showed that the expression levels of LINC01018 in liver are associated with traits of BMI and insulin sensitivity index (Supplementary Data 1); epigenetic marker and 3-D chromatin interaction analysis in human liver found that the corresponding GWAS-eQTL colocalized locus for LINC01018 contains both H3K27ac and H3K4me1 active enhancer markers and interacts with the genomic region of LINC01018 (Supplementary Data 2); human tissue expression profiling in GTEx dataset (Fig. 1b) as well as our qPCR results using human tissue cDNA panels (Fig. 3a) showed that the expression of LINC01018 is highly enriched in liver tissue. Although open reading frames were computationally detected on LINC01018 (Supplementary Fig. 1b), coding potential analysis using multiple algorithms (Supplementary Fig. 1c) and an in vitro translation assay (Fig. 3a) demonstrated that LINC01018 is non-coding. Interestingly, LINC01018 is robustly upregulated by fasting in humanized livers based on our RNA-seq analysis (Supplementary Data 5) and subsequent qPCR analysis (Fig. 3b). As regulation of gene expression in humans can sometime be strongly influenced by the genetic background, we also examined LINC01018 expression in humanized mice generated with human hepatocytes from a second independent donor and found that the upregulation of LINC01018 by fasting was well-maintained (Fig. 3b). The estimated average copy number of LINC01018 in human liver cells is ~74 (Supplementary Fig. 1d), and subcellular fractionation of humanized liver tissues showed LINC01018 is distributed in both of cytoplasm and nucleus with more in the cytoplasm (Supplementary Fig. 1e). Collectively, LINC01018 is a metabolic trait-associated, non-conserved, and liver-enriched human lncRNA that may function in fasting-related liver metabolic processes.

The aforementioned co-expression module analysis has placed LINC01018 in a module involved in diverse critical metabolic pathways in human liver (Fig. 1c, d and Supplementary Data 4). To pinpoint genes and pathways that specifically correlate with LINC01018, we next performed a focused lncRNA-mRNA correlation analysis. Using human liver RNA-seq data from GTEx, pairwise Pearson correlations were calculated between the expression of LINC01018 and all liver-expressed mRNAs. Then list of the top 300 correlated coding genes was used to define enriched KEGG pathways. As shown in Fig. 3c, this analysis found LINC01018 may function in fatty acid degradation, peroxisome and butanoate metabolism, corroborating the most significant pathways overrepresented by the LINC01018 co-expressed module M12 shown in Fig. 1c, d (Supplementary Data 4). These pathways become activated in the liver during fasting, a condition known to increase LINC01018 expression.

As most reported functional lncRNAs, including those we have previously characterized^10,11,28^ perform their functions by regulating gene expression, it is likely that LINC01018 might also modulate the expression of its correlated genes. Meanwhile, LINC01018 is robustly upregulated by fasting, suggesting it may contribute to the increased expression of these fasting-induced genes. Thus, we overlapped genes specifically correlated with LINC01018 in human livers with fasting upregulated human genes identified in humanized livers and defined them as potential LINC01018 target genes (Fig. 3c). To test if these genes can be regulated by LINC01018 in vivo, we used adenoviruses to express short hairpin RNAs (shRNAs) targeting LINC01018 in the humanized mice, and this strategy successfully reduced LINC01018 expression by 70% (Fig. 3d). Consequently, we found that while the expression levels of human ACOX1, ADH1B, EHHADH, and ACOX2 were not affected, knockdown of LINC01018 resulted in decreased expression of ADH1C, CYP4A11, and CYP4A22 in the ω-hydroxylation pathway of fatty acid metabolism, as well as BDH1 and ALDH5A1 in the butanoate metabolism pathway (Fig. 3d). We noticed that the expressions of NSUN2 and SRD5A1, the closest neighbor genes of LINC01018, were not affected by LINC01018 knockdown (Supplementary Fig. 1f), suggesting that LINC01018 unlikely acts in cis. Knockdown of LINC01018 using a second shRNA in humanized mice showed similar effects on the expression of these genes, supporting the regulatory effects we observed are specifically mediated by LINC01018 (Supplementary Fig. 1g). To examine whether the regulation of gene expression by LINC01018 is influenced by genetic background, we knocked down LINC01018 in humanized mice generated from hepatocytes of a second independent donor and found that the expression levels of ADH1C, CYP4A11, CYP4A22, BDH1, and ALDH5A1 in these mice were similarly decreased (Fig. 3e). Although it is very likely that lncRNAs might play unique roles in individuals with different genetic background, our data indicate the specific regulation of gene expression by LINC01018 defined by our strategy is likely independent of the effect of genetic background.

Taken together, these data suggest that LINC01018 regulates the expression of genes in fatty acid metabolism. It is a useful strategy to define potential target genes of a lncRNA by intersecting genes in lncRNA-associated top pathways and the regulated genes in an experimental condition where the lncRNA of interest is also regulated, such as fasting in our case. More importantly, our analyses here define putative target genes of human lncRNAs based on human data, which would allow for the identification of targets with direct human implications and also help avoid potential artifacts introduced by the humanized livers.

### The function of LINC01018 cannot be recapitulated in vitro

Cultured primary human hepatocytes (PHH) are widely used for studying the regulation of human hepatic genes, and we are interested in testing if the function of LINC01018 could be recapitulated in this in vitro system. Indeed, the relative expression of LINC01018 in cultured PHH were maintained at levels comparable to those in humanized livers. Since LINC01018-correlated genes in hepatic fatty acid degradation pathway are usually activated during fasting in a PPARα-dependent manner^29^, we reasoned that treatment of PHH with PPARα agonists might be able to induce the expression of genes in fatty acid degradation pathway and LINC01018. Consistent with previous reports^30^, treatment of PHH with PPARα agonist GW7647 robustly upregulated the expression of CYP4A11 and CYP4A22 (Supplementary Fig. 2a). However, no induction of LINC01018 expression was observed under this condition. Furthermore, common stresses showed no effect on the expression of LINC01018 in PHH (Supplementary Fig. 2b). Nevertheless, we tried to test the effects of LINC01018 knockdown in cultured PHH. While the same shRNA used in Fig. 3d successfully blocked the expression of LINC01018 by 70%, the expression of its target genes was not affected in PHH (Supplementary Fig. 2c). These results suggest that cultured PHH are not suitable to study the regulation and function of LINC01018 that we observed in humanized mice, highlighting the importance of an in vivo model for studying the function of human lncRNAs in metabolic regulation.

### HuR mediates the regulatory effects of LINC01018 in vivo

**Fig. 4:**
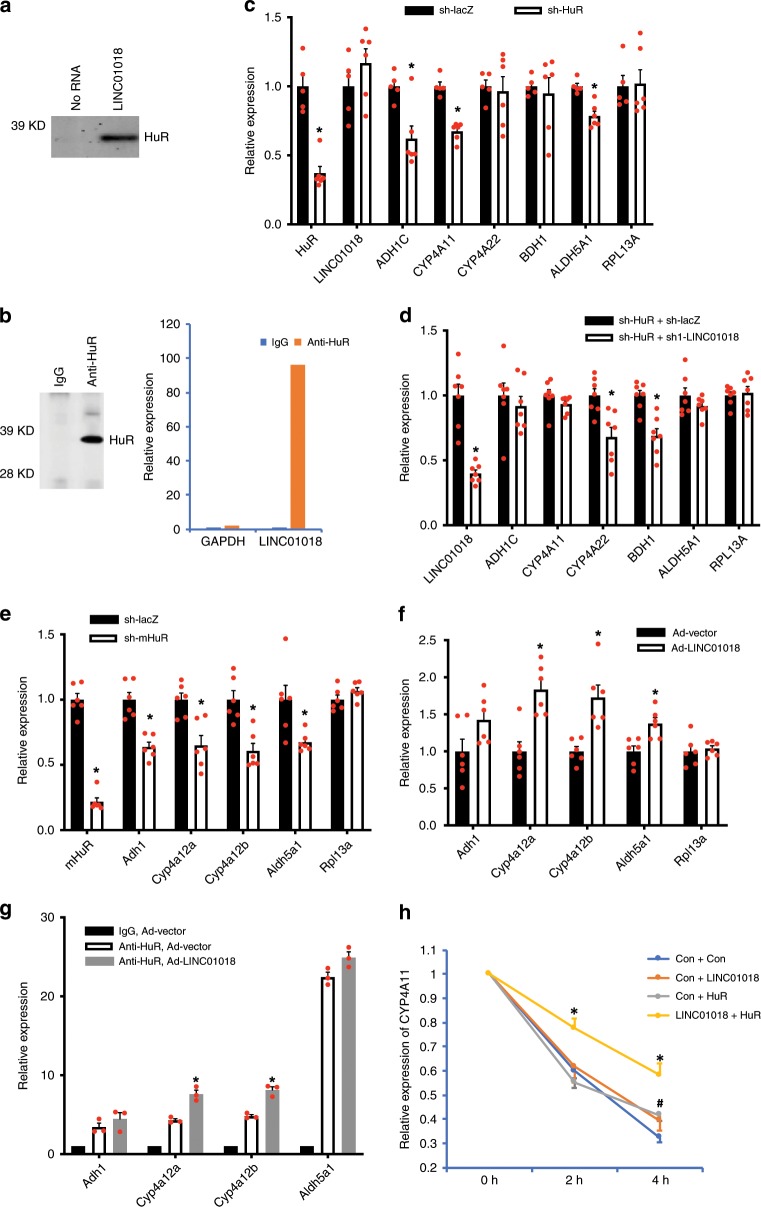
HuR mediates the regulatory effects of LINC01018. **a** Immunoblotting analysis of proteins in extract of humanized liver tissues that bind to biotinylated LINC01018 or a control using an anti-HuR antibody. **b** Left, anti-HuR immunoblotting analysis of proteins in immunoprecipitates of humanized liver tissues using an anti-HuR antibody. Right, Glyceraldehyde 3-phosphate dehydrogenase (GAPDH) and LINC01018 RNA levels in immunoprecipitates of humanized liver tissues using an anti-HuR antibody. **c**. Expression levels of human HuR and LINC01018 target genes in the livers of control (LacZ sh, *n* = 5) and HuR KD (HuR sh, *n* = 6) humanized mice after a 24 h food withdrawal. **d** Gene expression in livers of humanized mice receiving both control (LacZ shRNA) and HuR KD, or LINC01018 KD and HuR KD adenoviruses (*n* = 7 for each group). **e** Gene expression in livers of wild-type mice receiving control (LacZ shRNA) or mouse HuR KD adenoviruses (*n* = 6 for each group). **f** Gene expression in livers of wild-type mice receiving control or LINC01018 overexpression (OE) adenoviruses (*n* = 6 for each group). **g** Relative RNA levels in anti-HuR immunoprecipitates using liver tissue lysates of wild-type mice receiving control or LINC01018 OE adenoviruses (*n* = 3 for each group). Data in (**c**–**g**) represent mean ± SEM, *p < 0.05, two-tailed unpaired Student’s *t*-test. **h** Relative expression of human CYP4A11 in 293 A cells treated with actinomycin D for 0, 2 and 4 h. Data represent mean ± SEM of three independent experiments, *p < 0.05 for “LINC01018 + HuR” compared with “Con + Con”, ^#^*p* < 0.05 for “Con + HuR” compared with “Con + Con”, two-tailed unpaired Student’s *t*-test. Source data of (**a**–**h**) are provided in the Source Data file.

To explore the molecular mechanisms by which LINC01018 regulates gene expression in vivo, we performed lncRNA pulldown analysis to identify human proteins enriched by LINC01018. This strategy successfully identified several RNA-binding proteins which are involved in RNA metabolism (Supplementary Data 6). Among those, we are particularly interested in HuR, a well-studied RNA-binding protein that regulates mRNA stability and additional steps of RNA metabolism^31^. To validate the specific interaction between HuR and LINC01018, we confirmed the mass spectrometry results by immunoblotting using an anti-HuR antibody (Fig. 4a). We further performed RNA immunoprecipitation using the HuR antibody to determine the interaction between endogenous HuR and LINC01018 in liver tissues of humanized mice. As shown in Fig. 4b, LINC01018 is highly enriched in HuR immunoprecipitate while there is no enrichment of an abundant control transcript, Glyceraldehyde 3-phosphate dehydrogenase (GAPDH). These data support that LINC01018 specifically interacts with HuR in vivo.

Since HuR is a well-known posttranscriptional regulator of gene expression, we reasoned that if LINC01018’s regulatory function is mediated by HuR, the transcription of LINC01018 target genes should not be affected by LINC01018 knockdown. To test this, we performed chromatin immunoprecipitation (ChIP) analysis using an anti-RNA Polymerase II antibody to determine the transcriptional activity of human ADH1C, CYP4A11, CYP4A22, BDH1, and ALDH5A1 in humanized livers. While RNA Polymerase II was highly enriched at the transcription start sites of these genes, no changes in their enrichment were observed after knocking down of LINC01018 in humanized mice (Supplementary Fig. 3a), suggesting that LINC01018 exerts its regulatory effects through a post-transcriptional mechanism and HuR is possibly involved in this process. To directly test this possibility, we used adenoviruses to deliver shRNA specially targeting human HuR in humanized mice and then examined the expression of LINC01018 target genes. When over 60% of human HuR expression in the livers of humanized mice was suppressed, the expression levels of three out of the five LINC01018-regulated genes, ADH1C, CYP4A11 and ALDH5A1, were significantly decreased (Fig. 4c), suggesting that HuR may mediate some of the regulatory effects of LINC01018. To further test the functional dependency of LINC01018 on HuR, we simultaneously knocked down LINC01018 and human HuR in liver tissues of humanized mice. As shown in Fig. 4d, when the hepatic expression of human HuR is blocked, knockdown of LINC01018 showed no effects on the expression of ADH1C, CYP4A11, and ALDH5A1, while the effects on the expression of CYP4A22 and BDH1 are largely remained. These data support that LINC01018 functions, at least in part, in a HuR-dependent manner.

Although no mouse homolog of LINC01018 could be identified based on sequence similarity (Supplementary Fig. 1a), lncRNAs have been proposed to be conserved at additional levels including structure, function, or genomic locus^21^. To explore the possibility that a mouse homolog of LINC01018 might be transcribed from its syntenic region in the mouse genome, we searched for all potential uncharacterized transcripts in this region between Nsun2 and Ube2ql1 (Supplementary Fig. 3b) and determined their expression levels in mouse liver. Among the ten putative annotated lncRNAs, only two (Gm48819 and Gm18313) were marginally detectable in mouse liver. However, neither of them shows significant enrichment in HuR immunoprecipitation (Supplementary Fig. 3c), nor responses to fasting in mouse liver (Supplementary Fig. 3d) as LINC01018 does. Given their very low expression levels in mouse liver (both with estimated copy number/cell less than 0.5 vs 74 of LINC01018 in human hepatocytes), Gm48819 and Gm18313 are unlikely to be LINC01018’s mouse homologs.

As the mouse homologs of LINC01018 cannot be immediately identified or they might not exist at all, we sought to test whether the observed function of LINC01018-binding protein HuR, is conserved between human and mouse, and whether ectopic expression of LINC01018 regulates the activity of mouse HuR in regular mice. To this end, adenoviruses carrying shRNA targeting mouse HuR were delivered to regular mice and the expression levels of mouse homologs of human ADH1C, ALDH5A1, and CYP4A11 were determined. When more than 70% HuR expression in the mouse liver was suppressed, the expression levels of mouse Adh1, Aldh5a1, Cyp4a12a, and Cyp4a12b were significantly decreased (Fig. 4e), suggesting the regulation of LINC01018 target genes by HuR is conserved in mouse. Next, the adenoviruses expressing full-length cDNA of LINC01018 were delivered into regular mice to express LINC01018 in mouse livers to a level that is comparable to that in human liver tissues, and HuR immunoprecipitation assay clearly showed that LINC01018 can efficiently bind to mouse HuR (Supplementary Fig. 3e). More importantly, we found there was a substantial induction in the expression of mouse Cyp4a12a, Cyp4a12b and Aldh5a1 in liver tissues of LINC01018-expressing mice as compared to those in mice receiving control viruses (Fig. 4f), suggesting that LINC01018 could regulate HuR activity in mouse liver. To direct test if LINC01018 regulates HuR activity in mice, we performed HuR immunoprecipitation assay and found expression of LINC01018 significantly enhanced the interaction between HuR and mRNAs of Cyp4a12 isoforms in mouse liver (Fig. 4g). To clearly demonstrate the coordinated regulation of mRNA stability by LINC01018-HuR complex, we overexpressed human CYP4A11 with combinations of LINC01018 and HuR in HEK293A cells and performed actinomycin D treatment to determine the rate of RNA degradation. As shown in Fig. 4h, while overexpression of LINC01018 or HuR alone showed minimal effects on the stability of CYP4A11 mRNA, simultaneously expression both of them drastically slowed down the degradation of CYP4A11. These data further support that LINC01018 regulates the mRNA stability of its target genes by modulating the activity of HuR.

Finally, to further determine the pathophysiological relevance of LINC01018 in human, we analyzed human liver RNA-seq data related to non-alcoholic fatty liver disease (NAFLD), and found that the expression levels of LINC01018 are significantly decreased in the livers of NAFLD patients compared to those in healthy control subjects (Supplementary Fig. 4a). More interestingly, a low-carbohydrate dietary intervention in NAFLD patients significantly increased the expression of LINC01018 in the liver, which was accompanied by the upregulation of LINC01018 target genes (Supplementary Fig. 4b). These data suggest that LINC01018 could contribute to a protective metabolic profile in humans with fatty liver disease. Indeed, when challenged with a high sucrose diet which induces robust de novo lipogenesis in liver, regular mice expressing LINC01018 showed lower circulating levels of triglycerides and free fatty acids as compared with control mice (Supplementary Fig. 4c).

Taken together, these results suggested that LINC01018, a non-conserved human lncRNA, regulates a conserved function of HuR to modulate metabolic gene expression. Although a scenario where a lncRNA with similar function as LINC01018 exists in the mouse liver cannot be completely excluded, our data support that non-conserved human lncRNAs could potentially carry out human-specific regulation in energy metabolism.

## Discussion

Most human lncRNAs are currently considered non-conserved making it extremely challenging to identify the potentially functional ones and to further define their functions in vivo. Here, we develop a pipeline to simultaneously addresses both challenges. Using this pipeline, we have provided a proof of principle that one such lncRNA, LINC01018, regulates the expression of liver metabolic genes in vivo. We further demonstrated that LINC01018 interacts with HuR to exert its function. Intriguingly, although our observed regulatory effects of HuR on metabolic genes are conserved in mice and humans, LINC01018 is only expressed in humans, constituting an additional layer of fine-tuning mechanism. Thus, by establishing a fully integrated end-to-end experimental pipeline, our work opens up an avenue to evaluate the functional importance of non-conserved human lncRNAs and to uncover lncRNA-mediated mechanism in human biology.

The limited sequence conservation of human lncRNAs currently poses a major challenge in defining their function in human physiology. But as our understanding of lncRNA function is still rudimentary, we might not yet understand how lncRNAs are conserved, and lncRNA function could be conserved at a level that cannot be effectively assessed by current tools that are mostly designed for protein-coding genes. Thus, the percentage of lncRNAs that are functional could be far greater than those suggested by current sequence-based conservation analysis. Nonetheless, as we currently lack an effective strategy to use sequence information to identify functional lncRNAs, we have undertaken considerable effort to identify human lncRNAs that are likely to be functional and strive to provide as much functional inference as possible to ease their downstream analysis. We first systemically screen for liver-enriched lncRNAs that are regulated by eQTL-GWAS loci associated with a metabolic diseases. Liver function is highly relevant to the pathophysiology of these metabolic disorders and a number of liver-enriched lncRNAs have been shown to regulate various aspects of hepatic and systemic metabolism in mice^10–12,28^. Meanwhile, we performed additional analyses to enhance the confidence in the functionality of these trait-associated lncRNAs. For example, multiple lines of evidence generated from our analyses suggest that LINC01018 could play a role in metabolism including its regulation by a GWAS-eQTL pair with additional support by liver epigenetic markers and 3-D chromatin interactions, enrichment of expression in the liver, and correlated expression with genes involved in fatty acid metabolism pathways as well as its regulation by fasting. It should be noted that the regulatory information of these lncRNAs by metabolic milieus in vivo in humanized mice was obtained under a pure human genetic background and under well-controlled experimental conditions, which should be very instructive for the downstream in vivo functional analysis of lncRNAs. Combining these extensive analyses, we have identified a set of metabolic trait-associated human lncRNAs with multiple functional indicators, warranting the effort for focused functional analyses.

The most practical challenge for studying human lncRNAs is probably the lack of an experimental system to define their physiological function. Since most human lncRNAs are human- or primate-specific, they can often only be studied in human systems. However, most hepatic cell lines or primary hepatocytes derived from humans often exhibit substantial deviations from hepatocytes that reside in a natural physiological condition in the body. To address this challenge, we employed a liver-specific humanized mouse model to study human lncRNAs and have successfully identified the specific function of a non-conserved human lncRNA. Intriguingly, we cannot detect a similar functional impact of this lncRNA in primary hepatocytes, a phenomenon we and others have observed repeatedly in the process of characterizing mouse lncRNAs^11,12,28^. These findings collectively support that an in vivo system could be essential to define the biological role of non-conserved human lncRNA genes.

Of course, humanized liver is not human liver and clearly has its limitations. Similar to other organs, liver is composed of structured organization of multiple cell types but only hepatocytes are partially “humanized” in the current model. In this chimeric liver, the proportion of mouse hepatocytes or other cells could potentially affect the results. Additionally, the humanized mice are not suitable to study human lncRNAs whose functions require the human hormonal system. This model also has a limitation to study the early liver maturation, as it is derived only after mice reach to adult. It is well established that immunity and metabolism are highly interconnected^32^ but the mice for human hepatocyte engraftment are immune-deficient and are not suitable to study lncRNAs that function in this aspect. Presently, our humanized liver model is only an approximation of the human liver and might only be suitable to study certain aspects of human liver metabolism. However, this approximation is still worth the effort. Human hepatocytes in the humanized mice express thousands of human-specific lncRNAs many of which can only be maintained in the physiological environments in the liver. Additionally, we are mindful of experimental artifacts that could be induced by such an approximation and have took steps to mitigate such a possibility. For example, we have derived all our lncRNA targets from human data and restrained potential lncRNA-regulated genes to those that are supported by human data such as gene co-expression analysis based on GTEx data resource. Similar to any other model system, the liver-specific humanized mice have been and will be continuously improved to more reliably mimic human liver. Both hepatocytes and hematopoietic cells can be engrafted simultaneously to study processes that require interactions between hepatocytes and hematolymphoid cells^33^. Additional metabolic organs such as adipose and muscle can theoretically also be simultaneously humanized in the same mice which would establish a more complete system to study the role of lncRNAs in organ communication.

The number of non-conserved human lncRNAs has already exceeded that of mRNAs, and functional analysis of these lncRNAs could provide many fundamental insights into human physiology and disease. For example, two non-conserved human lncRNAs were recently shown to regulate adipocyte and cholesterol metabolism respectively in cell^17,18^. The current study takes a step further to demonstrate the physiological relevance of human-specific lncRNAs in an in vivo setting. GWAS studies routinely assign genomic loci to protein-coding genes and often ignore the effects of lncRNAs. We have shown here that a trait-associated human lncRNA modulates a metabolic pathway that could play a role in its associated disease. Further functional studies of additional trait-associated lncRNAs could provide critical insights into the genetic basis of common human diseases. Ultimately, a deeper genetic and molecular understanding of how non-conserved human lncRNAs shape gene expression and cell biology could translate into a better pathophysiological characterization of human diseases and more effective and personalized therapies.

## Methods

### RNA-seq analysis pipeline

The liver RNA-seq data for normal human population were downloaded from GTEx project. The liver RNA-seq datasets for 14 healthy controls and 15 non-alcoholic fatty liver disease (NAFLD) patients, and 7 NAFLD patients before and after low-carbohydrate dietary intervention were retrieved from SRP186450 and SRP126075 respectively in Sequence Read Archive (SRA). After quality control (FastQC: https://www.bioinformatics.babraham.ac.uk/projects/fastqc/) and trimming (TrimGalore: https://www.bioinformatics.babraham.ac.uk/projects/trim_galore/), fastq files were aligned to an index created using GRCh38 genome and the lncRNAKB annotation reference using HISAT2^34^. Sambamba sort^35^ was used to compress and sort the resulting sam files, and featureCounts^36^ from the Subread package^37^ was used to generate expression count levels for each sample. Outlier samples with low reads aligned (<1 million) were removed. Protein-coding genes with CPM^38^ <2 and non-coding genes with CPM <1 in half of samples were excluded from further analysis.

### Liver eQTL discovery for lncRNAs

Liver eQTL discovery was conducted using GTEx v7 RNA-seq data and WGS genotyping data (*n* = 118 samples)^39^. The GTEx RNA-seq data was quantified using our custom annotation described above. The genotyped samples are predominantly from individuals with European ancestry, but also includes those with African or Asian ancestry. The genotyping data was liftedOver from GRCh37 to GRCh38. Our pipeline closely followed the eQTL discovery pipeline used by the GTEx consortium (http://github.com/broadinstitute/gtex-pipeline). Expression data was TMM normalized^40^ and genes were filtered based on the cutoffs described above. Cis-QTL analysis was performed using FastQTL^41^ testing all SNPs within 1 Mb around the gene. The top three genotyping principal components (PCs), 15 Probabilistic Estimation of Expression Residuals (PEER)^42^ factors, sex, and genotyping platform were included as covariates in the analysis. Multi-allelic SNPs were discarded and the remaining SNPs were annotated using dbSNP v151 database^43^.

To address the reliability of lncRNA *cis*-eQTLs, a permutation strategy was used. We performed an adaptive permutation pass (between 1000 and 10,000 permutations) on the data in FastQTL. The same covariates and parameters as the initial *cis*-eQTL analysis were used to run the permutations. All lncRNA genes with an empirical permutation *p*-value ≤ 0.05 (*n* = 1701) were selected for further analysis. In addition, only SNP-lnc-eGene pairs with *cis*-eQTL *p*-value ≤ 5 × 10^−4^ (*n* = 146,062) were further chosen for the SMR analysis.

### Colocalization analysis of GWAS and eQTL signals

SMR (Summary-data-based mendelian randomization) and HEIDI (Heterogeneity in dependent instruments) methods implemented in SMR program were used to test the association between gene expression and traits and the colocalization of GWAS and eQTL signals. Particularly, HEIDI uses multiple SNPs in a cis-eQTL region to distinguish pleiotropy from linkage, and a pHEIDI > 0.05 suggests non-heterogeneity, thus colocalized. Briefly, summary GWAS data for major metabolic traits available in the six consortia for cardiometabolic GWAS meta-analyses were downloaded (Supplemental Data 1), and formatted into.ma format as specified on the CNS genomics’ website (http://cnsgenomics.com/software/smr/). The eQTL summary data consist of 146,062 *cis*-eQTLs (*p*-value ≤ 5 × 10^−4^) in 1694 lncRNAs selected with an empirical permutation *p*-value ≤ 0.05. Results from FastQTL were formatted into BESD format. Analysis was then conducted separately using GWAS meta-analyses summary data for each of the 29 cardiometabolic traits (Supplementary Data 1) using a default cis window of 2000kb and peQTL set to 5 × 10^−4^ for top SNPs, with the 1000 Genomes data used as a ref. ^44^. Considering the limited statistical power of our eQTL calculation due to the relatively small number of liver samples from GTEx, we initially kept lncRNAs from SMR analysis with nominal pSMR < 0.05 but pHIEDI > 0.05 and used additional downstream analyses descried below to further select functional lncRNAs.

### Epigenetic marker and Hi-C chromatin interaction analysis

Reads of 5 runs of human liver Hi-C sequencing data from a single sample (SRX641267)^45^ were analyzed using Hi-C Pro software^46^ using the default criteria. Valid pairs of genomic fragments with spatial interactions were retrieved. H3K27ac (ENCSR678LND and ENCSR230IMS) or H3K4me1 (ENCSR489DUV, ENCSR218ZMU, ENCSR642HII, and ENCSR111OHT) histone mark enrichment from ChIP-seq assays of human livers were downloaded from the ENCODE. To find evidence of interaction between eQTL SNPs, H3K27ac/H3K4me1 and lncRNAs, we required that one fragment of the Hi-C pair contained an eQTL SNP identified in our target pool as well as overlapped with one or more peaks of H3K27ac/H3K4me1 enrichment and the other fragment of the Hi-C pair overlapped one of the 726 lncRNA genes by at least one basepair. 320 genes were found to have support from at least one such Hi-C pair.

### Conservation analysis

Detailed method could be found at the website of lncRNAKB (http://psychiatry.som.jhmi.edu/lncrnakb/methods.php). Briefly, conservation of exons for lncRNAs was analyzed using the bigWigAverageOverBed and the cons30way (hg38) track both downloaded from the UCSC genome browser. This track shows multiple alignments and measurements of evolutionary conservation for 30 vertebrate species. An exon-level BED file was created using the lncRNAKB GTF annotation file for lncRNAs. We merged overlapping exons within transcripts to avoid counting conservation scores of overlapping base pairs more than once. For each exon, the bigWigAverageOverBed function calculates the average conservation score across all base pairs. A mean score was then calculated from all exons for each lncRNA gene. The average mean conservation score for the 726 trait-associated lnc-eGenes is 0.12.

### Liver enrichment

Liver enriched genes were defined as those with a liver expression z-score > 2. All available samples in GTEx were re-analyzed using our RNA-seq analysis pipeline. For each tissue, average CPM was calculated across all samples and tissue specific z-scores for each gene were calculated.

To test whether our trait-associated lnc-eGenes have stronger liver-enriched expression in general, the mean liver z-score was calculated across all trait-associated lnc-eGenes. This process was then repeated 10,000 times with randomly sampled lists of liver-expressed genes with replacement of the same size as our list of trait-associated lnc-eGenes. For each resampling, the mean z-score was collected to create an empirical liver z-score distribution. The mean z-score for the trait-associated lnc-eGenes was compared to this distribution to obtain an empirical *p*-value for liver enrichment.

### Functional prediction of lncRNAs using a network approach

To identify the potential function of the 726 lncRNAs identified using the SMR analysis, CEMitools R package^26^ was used to automatically implement gene co expression network analysis and perform pathway enrichment using the KEGG pathways downloaded from MSigDB^47^. The clusters identified were further filtered based on two criteria: (1) Presence of at least one lncRNA identified from liver-eQTL-GWAS analysis (lnc-eGenes). (2) Genes in the cluster enriched in at least one KEGG pathway with *q* value < 0.05. This resulted in identification of 29 modules/gene clusters.

### Focused lncRNA-mRNA correlation analysis

To find potential specific target genes for an interested lnc-eGene, human liver RNA-seq data from GTEx were used for a focused lncRNA-mRNA correlation analysis. Briefly, pairwise Pearson correlations were calculated between lnc-eGene expression and mRNA expression for all liver-expressed coding genes (cpm ≥ 2 in half of the samples). KEGG pathway enrichment was calculated for the top 300 correlated coding genes, and the genes enriched in the top 3 pathways were considered as potential candidates for lnc-eGene target genes.

### RNA-seq analysis of livers from humanized mouse

Human annotation from lncRNAKB was combined with the Refseq mouse annotation to make a hybrid genome annotation of human and mouse for analyzing RNA-seq data of the chimeric livers from humanized mice. Contigs for each annotation were first prefixed with “human_” and “mouse_” depending on the source organism. We also followed the same procedure and generated the combined FASTA file for indexing. Eight humanized mouse RNA-seq samples (four from fasting mice; four from fed mice) were processed using our RNA-seq pipeline. Once the expression table was generated by featureCounts, human genes were separated for further downstream analyses. The DESeq2 R package^48^ was then used to calculate differentially expressed genes between fed and fasted mice.

### Animal experiments

All animal experiments were performed in accordance and with approval from the NHLBI Animal Care and Use Committee or the Animal Care Committee of the Central Institute for Experimental Animals (CIEA). Animal data were excluded from experiments based on pre-established criteria of visible abnormal liver structure during sample harvest or other health issues such as fighting wounds or infections. According to the variability of metabolic parameters, group size was determined based on previous studies using similar assays within the laboratory and pilot experiments. Experimenters were not blinded to treatment group.

TK-NOG mice, in which a herpes simplex virus type 1 thymidine kinase (TK) transgene under a mouse albumin promoter is expressed within the liver of highly immune-deficient NOG mice, were obtained from Taconic Biosciences. The TK converts an antiviral medication ganciclovir (GCV) into a toxic product that allows selective elimination of TK positive cells in vivo. The cryopreserved primary human hepatocytes were obtained from Thermo Fisher Scientific (first donor) or BioIVT (second donor). The humanized TK-NOG mice were prepared as previously described^27^. Briefly, The TK-NOG mice at 8–9 weeks old received an i.p. injection of GCV at a dose of 25 mg/kg. One week later, 50–μl volume of 1 × 10^6^ human primary hepatocytes suspended in HBSS solution were transplanted via intra-splenic injection. 8–12 weeks after transplantation, the serum human albumin in the mice were measured as an index of the extent of human hepatocytes replacement. Humanized TK-NOG mice with serum human albumin levels above 0.5 mg/ml were used for experiments, in which human hepatic genes could be reliably detected by q-PCR. For the fasting study, humanized mice were produced and the experiment was carried out at CIEA. Humanized mice for the rest of the study were produced and analyzed at NHLBI. For the fasting study, humanized TK-NOG mice were either allowed free access to food or subjected to a twenty-four hours food withdrawal before tissue harvest.

Male C57BL/6 (B6) mice were purchased from Jackson Laboratory at 8 weeks of age, and housed 3–5 mice per cage with free access to water and normal chow diet (NIH-31), and animals were acclimatized to the housing for 10–14 days before experiments. Groups of co-housed mice were randomly assigned to experimental groups with age and weight in accordance between groups. For high sucrose diet feeding experiments, 3 days after adenovirus injection, mice were fed with high sucrose diet (Research Diets Inc, Cat: D08020801) for 4 days and then plasma and liver tissues were harvested for biochemistry analysis. Triglyceride and free fatty acid levels were determined using kits from BioAssay Systems.

### Primary human hepatocytes

The cryopreserved primary human hepatocytes were obtained from Thermo Fisher Scientific (first donor), BioIVT (second donor) or Lonza (for the fasting study). Most of our experiments used the hepatocytes from Thermo Fisher Scientific (first donor), except explained elsewhere in the text (second donor or for the fasting study). For in vitro culturing of primary human hepatocytes, cell culture medium was prepared and the standard procedure was followed as recommended by manufacturers. For GW 7647 (Sigma) treatment, overnight plated primary human hepatocytes (serum free) were first incubated in William Medium E for four hours and followed by control (DMSO) or 10 μM GW 7647 treatment for another four hours. For stress treatment, overnight plated primary human hepatocytes (serum free) were treated with 5% fetal bovine serum, or 100 μM H_2_O_2_ for 4 h. For heat shock treatment, cells were first cultured at 42 °C for 30 min and then recovered at 37 °C for 4 h before harvest. For checking gene expression with knocking down of LINC01018, 4 h after plating, human primary hepatocytes were infected with sh-lacZ or sh1-LINC01018 adenovirus at MOI of 10 for 48 h, and then the RNA were extracted from these cells and quantitative Real-time PCR were performed to determine gene expressions.

### Adenovirus production and in vivo adenovirus administration

The shRNAs for LINC01018, human HuR and mouse HuR were designed using the following sequences (LINC01018 shRNA1: CCTTAAACTTGTACCACTT; LINC01018shRNA2: GCAAGAAGACCCAGCTATT; human HuR shRNA: GGCTTTGTGACCATGACAA; mouse HuR shRNA: GGTTTGGGCGAATCATCAA). The hairpin template oligonucleotides were synthesized by Integrated DNA Technologies and were subsequently cloned into the adenovirus vector of the pAD/Block-it system (Invitrogen) according to the manufacturer’s protocols. Overexpression construct of LINC01018 was generated by PCR-amplifying the sequence of NR_024424.2 using human liver cDNA sample. The sequence was subsequently cloned into pAdv5 adenovirus vector for virus packaging. Adenoviruses were amplified in HEK293A cells (Invitrogen) and purified by CsCl gradient centrifugation. Purified viruses were desalted with PD10 columns (GE Healthcare Life Sciences) and titered with Adeno-X Rapid Titer Kit (Clontech). Adenoviruses were delivered into mice intravenously at 5 × 10^8^ pfu/mouse for overexpression, 1–2 × 10^9^ pfu/mouse for knocking down experiments. In the case of double-knockdown experiments, two viruses of equal titer were first mixed, then each mouse received 2 × 10^9^ pfu total virus. After seven days, tissue samples were harvested for further analysis.

### RNA pull-down and mass spectrometry analysis

Biotin-labeled RNAs were in vitro transcribed using the Biotin RNA Labeling Mix and T7 RNA polymerase (Ambion) and purified with the RNeasy Mini Kit (Qiagen) on-column digestion of DNA. The humanized liver tissue lysates were freshly prepared by homogenizing frozen liver tissues using a dounce homogenizer with 15-20 strokes in ProteaPrep Zwitterionic Cell Lysis Buffer supplemented with RNaseOUT, Protease/Phosphatase Inhibitor Cocktail, Panobinostat, and Methylstat. The BcMag Monomer Avidin Magnetic Beads (Bioclone) were first prepared in accordance with manufacturer’s instructions and then immediately subjected to RNA (20 μg) capture in RNA capture buffer (20 mM Tris-HCl [pH 7.5], 1 M NaCl, and 1 mM EDTA) for 30 min at room temperature with agitation. The RNA-captured beads were washed once with NT2 buffer (50 mM Tris-HCl [pH 7.4], 150 mM NaCl, 1 mM MgCl_2_, and 0.05% NP-40) and incubated with 30 mg cell lysates diluted in NT2 buffer supplemented with 50 U/ml RNaseOUT, 2 mM dithiothreitol, 30 mM EDTA, and 0.02 mg/ml Heparin for 2 h at 4 °C with rotation. The RNA-binding protein complexes were washed sequentially with NT2 buffer (twice), NT2 high-salt buffer containing 500 mM NaCl (twice), NT2 high-salt buffer containing 1 M NaCl (once), NT2-KSCN buffer containing 750 mM KSCN (twice), and PBS (once) for 5 min at 4 °C and eluted by 2 mM D-biotin in PBS. Samples (one sample for no RNA control and one sample for LINC01018 pull-down) were sequentially reduced with 10 mM Tris (2-carboxyethyl) phosphine hydrochloride, alkylated with 20 mM chloroacetamide, and then digested with trypsin at 37 °C overnight. The resulting peptides were desalted with ZipTip (Merck Millipore, MA), and subsequently vacuum-dried. Samples were then reconstituted with 0.1% formic acid, and analyzed in Data-Dependent Acquisition (DDA) mode on an Orbitrap Lumos (Thermo Fisher Scientific, San Jose CA) based nanoLCMS system. Peptides were separated on a 50 cm long EasySpray PepMap RSLC C18 column (Thermo Fisher Scientific) with a linear gradient of 5–30% acetonitrile delivered in 40 min. Precursor ions were first surveyed in 375–1500 Da mass range at 120 k resolution and 4e5 automatic gain control (AGC). They were sequentially fragmented with higher-energy collisional dissociation (HCD) at 30% energy. And fragment spectra were recorded at 30 k resolution and 5e4 AGC. The resulting LCMS data were searched against SwissProt Human database using Mascot (v2.6, Matrix Science Inc. Boston MA) via Proteome Discoverer 2.2 platform (Thermo Fisher Scientific). Search parameters are 10ppm and 0.02 Da mass tolerance for precursors and fragment ions, respectively, two tryptic miscleavages, and cysteine carbamidomethylation as fixed modification and methionine oxidation variable. Proteins and peptides were filtered with 1% false discovery rate (FDR) cutoff.

### **RNA immunoprecipitation** (RIP)

To prepare liver tissue lysates, frozen liver tissues were homogenized using a dounce homogenizer with 15–20 strokes in RIP buffer (150 mM NaCl, 20 mM Tris pH 7.4, 1 mM EDTA, 0.5% Triton X-100 with Protease/Phosphatase Inhibitor Cocktail and RNaseOUT). For each RIP, 10 μg rabbit IgG or HuR -RIP Validated Antibody (MilliporeSigma) were first incubated with 30 μl washed Dynabeads® Protein G in 300 μl RIP buffer supplemented with 0.2 mg/ml BSA, 0.2 mg/ml Heparin and 0.2 mg/ml EcoRI tRNA for one hour. Then the antibody coupled beads were added to 5 mg liver tissue lysates diluted in 500 μl RIP buffer and incubated for 3 h at 4 °C with gentle rotation. Beads were washed briefly five times with RIP buffer. At the final wash, one fifth of beads were used for protein analysis and the rest of beads were resuspended in 1 ml of Trizol for RNA extraction. Co-precipitated RNAs were isolated and analyzed by RT-PCR. The expression levels were normalized with those from input, and then the fold enrichments were compared with IgG control.

### ChIP analysis

ChIP assays of frozen liver tissue were performed using a Simple ChIP Enzymatic Chromatin IP kit (Cell Signaling Technology) according to the manufacturer’s protocol. Immunoprecipitation was performed using RNA Pol II ChIP validated antibody (MilliporeSigma, Cat: 17-620) or with rabbit IgG as a negative control. The DNA in each ChIP were determined by quantitative real-time PCR analysis using human-specific primers amplifying the genomic sequences covering the transcriptional start sites of genes (Supplementary Data 7). The relative enrichment by RNA Pol II was calculated by normalized the amount of ChIP DNA to input DNA and compared with IgG control as fold enrichment.

### RNA extraction and quantitative real-time PCR analysis

Total RNA was isolated from liver tissues or cells using Trizol reagent (Invitrogen). After Turbo DNA-free DNase treatment (Ambion), reverse transcription was carried out with SuperScript® III First-Strand Synthesis system (Invitrogen) using 1 µg of RNA. Quantitative real-time RT-PCR was performed on a ViiA™ 7 Real-Time PCR System (Applied Biosystems Inc.) The PCR program was: 2 min 30 s at 95 °C for enzyme activation, 40 cycles of 15 s at 95 °C, and 1 min at 60 °C. Melting curve analysis was performed to confirm the real-time PCR products. For detecting the expression of human genes in humanized liver samples, human-specific primers were designed and quantitation were normalized to human 16S rRNA levels. For detecting the expression of mouse genes in wild-type mice, 18S rRNA was used as the internal control. The full primer sequences used are provided in the Supplementary Data 7.

### Immunoblotting

For Immunoblotting analyses, the cells and tissues were lysed in 1% SDS lysis buffer containing phosphatase inhibitors (Sigma) and a protease inhibitor cocktail (Roche). The lysate was subjected to SDS–PAGE, transferred to polyvinylidene fluoride (PVDF) membranes, and incubated with the primary antibody followed by the fluorescence conjugated secondary antibody (LI-COR). The bound antibody was visualized using a quantitative fluorescence imaging system (LI-COR). The HuR Antibody was purchased from MilliporeSigma (Cat: 03-102, 1:1000 dilution for immunoblotting).

### Cytosol/nuclear fractionation

Humanized liver tissues were homogenized in nuclear isolation buffer (250 mM sucrose, 10 mM Tris-HCI, pH 7.4, 10 mM KCL, 1.5 mM MgCL2, 1 mM EDTA, 1 mM EGTA) with Protease/Phosphatase Inhibitor Cocktail and RNaseOUT. The lysate was centrifuged at 1000 × *g* for 3 min to pallet nuclear and the supernatant was used as cytosol fraction. The nuclear pallet was briefly washed with nuclear isolation buffer containing 0.1% NP-40 before RNA extraction. The relative expression of genes in cytosol and nuclear fraction was calculated by the total amount and set the cytosol fraction as 1.

### In vitro translation analysis

The in vitro translation analysis was performed using TnT® Quick Coupled Transcription/Translation System (Promega). Briefly, a T7 promoter was attached to 5-prime end of LINC01018 DNA by PCR amplification. Then the purified PCR products were used as templates to perform in vitro translation analysis by following manufacture’s protocol. Biotinylated lysine was used to label the newly synthesized proteins which were visualized by using IRDye Streptavidin (LI-COR). The open reading frame of yellow fluorescent protein (YFP) was used as a positive control.

### RNA stability analysis in 293A cells

293A cells were cultured under standard conditions. The full length of human CYP4A11 (NM_000778.4) and LINC01018 (NR_024424.2) were cloned into pAdv5 vector (Invitrogen) for expression in 293A cells. Empty pAdv5 vector was used as control for pAd-LINC01018. Vectors expressing human HuR (Cat: RC201562) and control (Cat: PS100001) were purchased from Origene. 24 h after transfection, 293A cells were treated with 0.5 μg/ml actinomycin D for 0, 2 and 4 h. RNA was extracted and RT-PCR were performed to determine the relative expression of CYP4A11 (normalized to time point 0 h) in each group.

### Reporting summary

Further information on research design is available in the Nature Research Reporting Summary linked to this article.

## Supplementary information

Supplementary Information

Reporting Summary

Description of Additional Supplementary Files

Supplementary Dataset 1

Supplementary Dataset 2

Supplementary Dataset 3

Supplementary Dataset 4

Supplementary Dataset 5

Supplementary Dataset 6

Supplementary Dataset 7

## Data Availability

A reporting summary for this Article is available as a Supplementary Information file. The source data underlying Figs. 2c, 3a–e, and 4a–h, and Supplementary Figs. 1f, g, 2a, c, and 4c are provided as a Source Data file. RNA-seq dataset “Fasting Fed RNA sequencing experiment of mice with humanized livers” (GSE126587) can be assessed at GEO. Dataset “LINC01018 Pulldown LCMS protein identification” (identifier PXD015791) can be accessed at ProteomeXchange via the PRIDE database. All data is available from the corresponding author upon reasonable request.

## Source Data

Source Data
