# Supplementary material for: In vivo functional analysis of non-conserved human lncRNAs associated with cardiometabolic traits
Source: Nat Commun. 2020 Jan 2;11:45. doi: 10.1038/s41467-019-13688-z (PMC6940387; doi:10.1038/s41467-019-13688-z)
Supplement: Supplementary file 1 — Supplementary Information [file 41467_2019_13688_MOESM1_ESM.pdf]

a

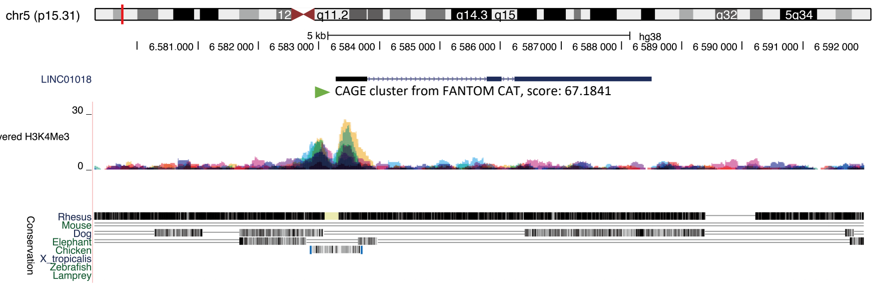

b

Label	Strand	Frame	Start	Stop	Length (nt | aa)
ORF1	+	1	1336	1428	93 | 30
ORF2	+	2	2447	2542	96 | 31
ORF3	+	3	2661	2765	105 | 34
ORF4	+	2	2093	2206	114 | 37
ORF5	+	2	2741	2863	123 | 40
ORF6	+	2	2582	2710	129 | 42
ORF7	+	2	32	172	141 | 46
ORF8	+	3	2277	2447	171 | 56
ORF9	+	2	905	1084	180 | 59
ORF10	+	1	511	750	240 | 79
ORF11	+	3	1902	2162	261 | 86
ORF12	+	2	1340	1600	261 | 86
ORF13	+	3	78	470	393 | 130

c

CNCI analysis

ID	Index	Score
LINC01018	noncoding	-0.0188416

CPC2 analysis

ID	Label	Coding probability
LINC01018	noncoding	0.0589835

PhyloCSF anlysis

ID	Interpretation	PhyloCSF score
LINC01018	noncoding	10.7511

d

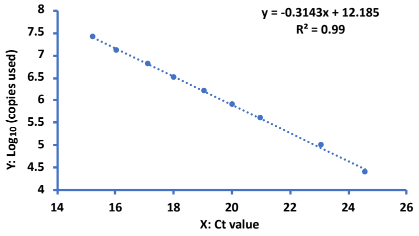

Donor	Ct value	Log_{10} (copies used)	Copies used	Cells used	Copies/cell
1	24.7	4.4142	25957	325	80
2	23.4	4.8452	70009	363	193
3	25.0	4.3335	21551	320	67
4	26.7	3.8032	6357	267	24
5	24.5	4.4796	30173	327	92
6	25.6	4.1295	13474	510	26
7	25.1	4.3108	20457	576	36
					74 (average)

e

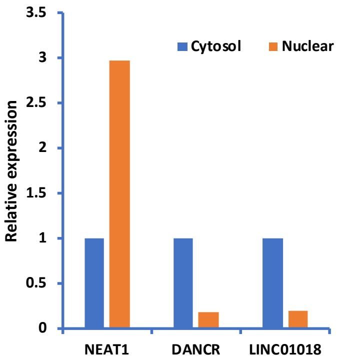

f

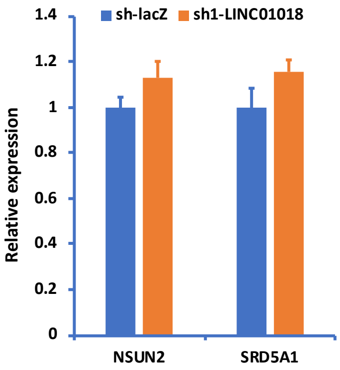

g

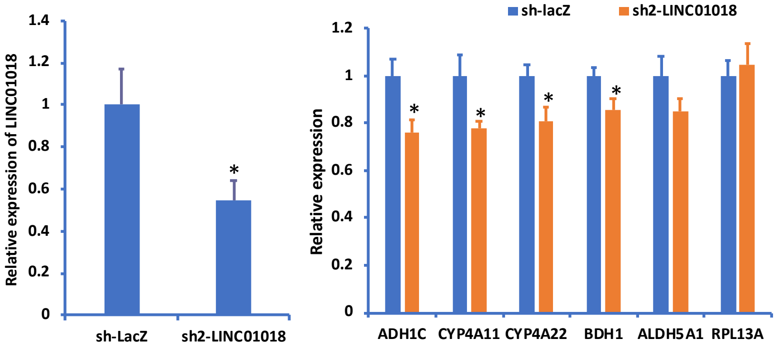

Supplementary Figure 1: Characterization of LINC01018. a. Gene structure of LINC01018 and relevant information of CAGE peaks at transcription start site, chromatin marks and conservation. **b.** Potential peptides encoded by LINC01018 as determined by ORF finder. **c.** Coding potential analysis of LINC01018 using CNCI, CPC2 and PhyloCSF tools. **d.** Quantification of LINC01018 copy number in freshly thawed primary human hepatocytes. LINC01018 cDNA concentrations were quantified with a standard curve prepared by PCR amplification of a serial dilution of pAd-LINC01018 plasmid. **e.** Levels of LINC01018 in cytosolic, or nuclear fractions of samples pooled from four humanized livers. LncRNA NEAT1 and DANCR were used as markers for nuclear and cytosolic fractions respectively. **f.** Expression levels of LINC01018 neighboring genes in the livers of control and LINC01018 knockdown (KD) humanized mice received shRNA adenoviruses for LacZ (LacZ sh, n=8) and LINC01018 (LINC01018 sh1, n=8) respectively. **g.** Gene expression in the livers of control (LacZ sh, n=6) and LINC01018 KD (LINC01018 sh2, n=6) humanized mice after a 24 hr food withdrawal. Data in (f, g) represent mean ± SEM, *p<0.05, two-tailed unpaired Student's *t*-test. Source data of (f, g) are provided in the Source Data file.

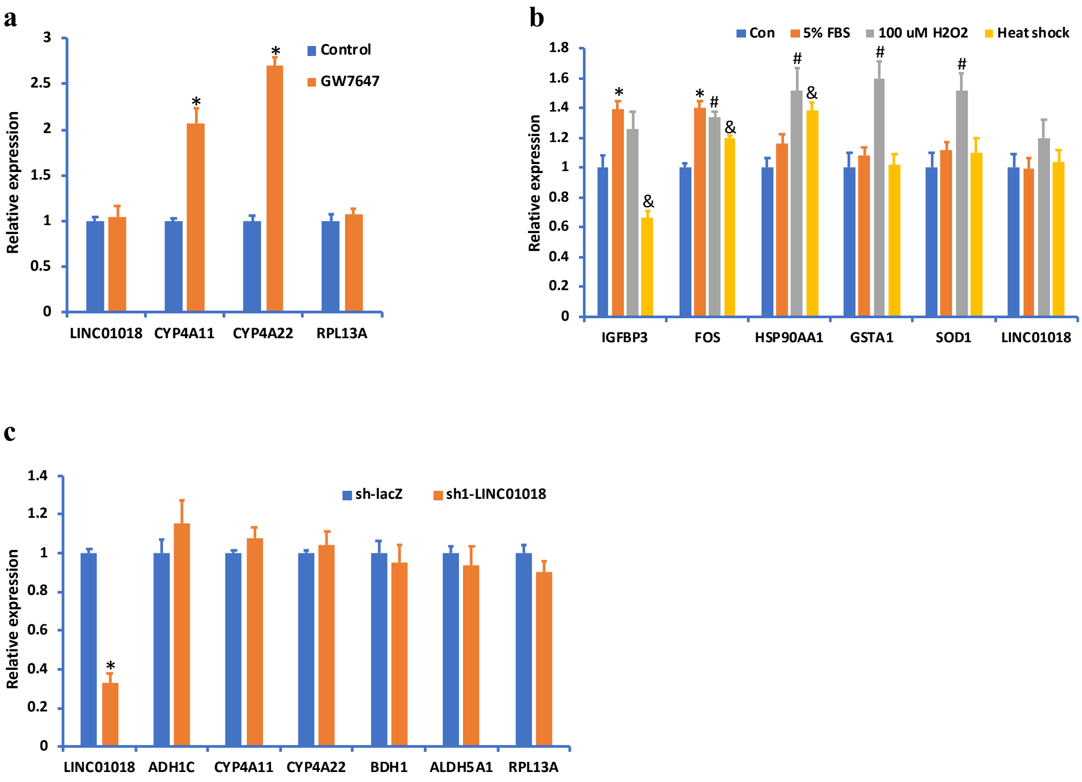

Supplementary Figure 2: The function of LINC01018 cannot be recapitulated *in vitro* **a.** Gene expression in cultured primary human hepatocytes treated with control or GW7647 (n=3 for each group). **b.** Gene expression in cultured primary human hepatocytes treated with control, 5% FBS, 100 μM H2O2 or heat shock (n=3 for each group). Data represent mean ± SEM, *, #, &, $p<0.05$ for 5% FBS, 100 μM H2O2 and heat shock compared with control respectively, two-tailed unpaired Student's *t*-test. **c.** Gene expression in cultured primary human hepatocytes infected with adenoviruses carrying lacZ shRNA or LINC01018 shRNA (n=3 for each group). Data in (**a, c**) represent mean ± SEM, *$p<0.05$, two-tailed unpaired Student's *t*-test. Source data of (c) are provided in the Source Data file.

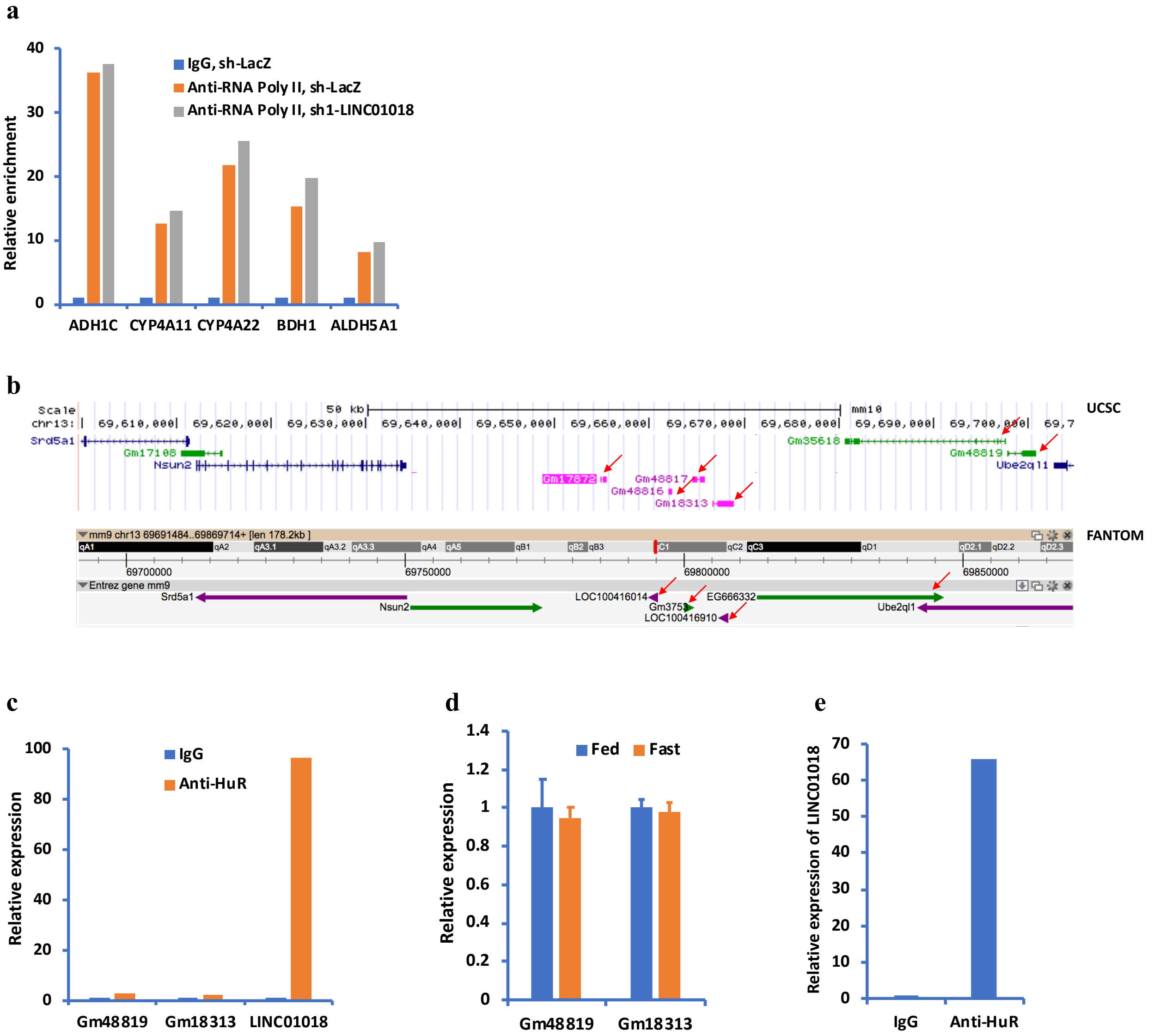

Supplementary Figure 3: No mouse homolog of LINC01018 is identified. a. Chromatin immunoprecipitation of liver tissues of control and LINC01018 KD humanized mice using an anti-RNA poly II antibody. Samples were pooled from three mice of each group. **b.** All annotated genes from UCSC (top panel) and FANTOM (bottom panel) between mouse Nsun2 and Ube2ql1 (syntenic region of human LINC01018) were pointed by red arrows. **c.** Relative expression of Gm48819 and Gm18313 in anti-HuR immunoprecipitates prepared from liver tissue lysates of wild-type mice. The relative expression of LINC01018 in anti-HuR immunoprecipitates prepared from liver tissue lysates of humanized mice (Fig. 4b) was also presented for comparison. **d.** Relative expression of Gm48819 and Gm18313 in liver tissues of wild-type mice subjected to fed or a 24-hour fasting (n=6 for each group). Data represent mean ± SEM. **e.** LINC01018 RNA levels in anti-HuR immunoprecipitates using liver tissue lysates of wild-type mice receiving LINC01018 OE adenoviruses.

a

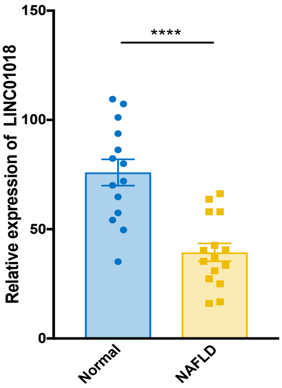

b

Gene	log2FoldChange (after/before)	p value
LINC01018	0.84013049	0.004164158
CYP4A11	1.058736261	5.79E-09
CYP4A22	0.822729278	1.17E-06
BDH1	0.251173	0.032864212
ALDH5A1	0.946965481	1.25E-05

c

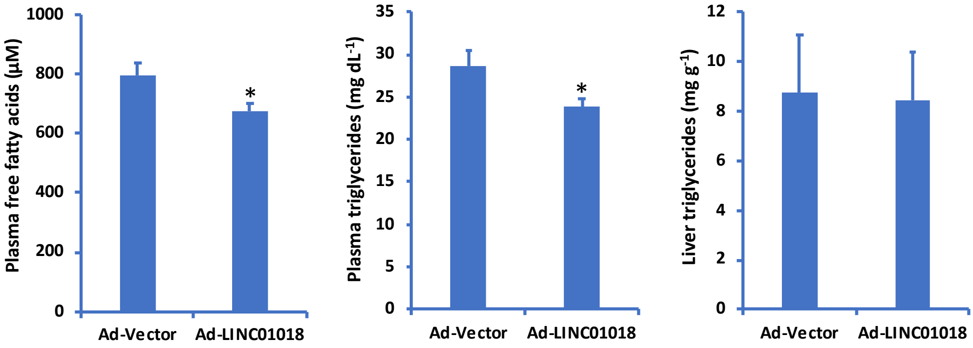

Supplementary Figure 4: LINC01018 functions in fatty acid metabolism. a. Relative expression of LINC01018 (as determined by CPM from RNA-seq analysis) in human liver tissues from Non-alcoholic fatty liver disease (NAFLD) patients (n=15) or health controls (n=14). Data represent mean ± SEM and the expression of each individual was plotted, ****, $p < 0.0001$, two-tailed unpaired Student's *t*-test. **b.** Expression fold changes of LINC01018, CYP4A11, CYP4A22, BDH1 and ALDH5A1 in human livers of NAFLD patients after/before low carbohydrate diet intervention (n=7). P value was calculated by paired Student's *t*-test. **c.** Plasma fatty acids levels (left), triglycerides levels (middle) and hepatic triglyceride contents (right) in LINC01018 expressing or control mice challenged with high sucrose diet (n=7 for each group). Data represent mean ± SEM, *$p<0.05$, two-tailed unpaired Student's *t*-test. Source data of (c) are provided in the Source Data file.
