# Supplementary material for: In vivo functional analysis of non-conserved human lncRNAs associated with cardiometabolic traits
Source: Nat Commun. 2020 Jan 2;11:45. doi: 10.1038/s41467-019-13688-z (PMC6940387; doi:10.1038/s41467-019-13688-z)
Supplement: Supplementary file 2 — Reporting Summary [file 41467_2019_13688_MOESM2_ESM.pdf]

nature research

Corresponding author(s): Haiming Cao

Last updated by author(s): Oct 20, 2019

Reporting Summary

Nature Research wishes to improve the reproducibility of the work that we publish. This form provides structure for consistency and transparency in reporting. For further information on Nature Research policies, see Authors & Referees and the Editorial Policy Checklist .

Statistics

For all statistical analyses, confirm that the following items are present in the figure legend, table legend, main text, or Methods section.

n/a	Confirmed	
☐	☒	The exact sample size (n) for each experimental group/condition, given as a discrete number and unit of measurement
☐	☒	A statement on whether measurements were taken from distinct samples or whether the same sample was measured repeatedly
☐	☒	The statistical test(s) used AND whether they are one- or two-sided<br>*Only common tests should be described solely by name; describe more complex techniques in the Methods section.*
☐	☒	A description of all covariates tested
☐	☒	A description of any assumptions or corrections, such as tests of normality and adjustment for multiple comparisons
☐	☒	A full description of the statistical parameters including central tendency (e.g. means) or other basic estimates (e.g. regression coefficient) AND variation (e.g. standard deviation) or associated estimates of uncertainty (e.g. confidence intervals)
☐	☒	For null hypothesis testing, the test statistic (e.g. F, t, r) with confidence intervals, effect sizes, degrees of freedom and P value noted<br>*Give P values as exact values whenever suitable.*
☒	☐	For Bayesian analysis, information on the choice of priors and Markov chain Monte Carlo settings
☐	☒	For hierarchical and complex designs, identification of the appropriate level for tests and full reporting of outcomes
☐	☒	Estimates of effect sizes (e.g. Cohen's d, Pearson's r), indicating how they were calculated

Our web collection on statistics for biologists contains articles on many of the points above.

Software and code

Policy information about availability of computer code

Data collection	Raw RNA-seq data of livers from Humanized Mouse were generated and collected following the protocol of Illumina Hiseq3000.
Data analysis	Bedtools for building a combined human annotation, Crossmap for lifting over annotations and genotyping files from build 37 to build 38, Trimgalore and FastQC for RNA-seq quality control, HISAT2 for RNA-seq alignment, Sambamba for sam file manipulation, Subread for RNA-seq quantification, vcftools and plink for genotyping data reformating, EISENSOFT for conducting Principal Component Analysis on genotyping data, EdgeR for RNA-seq filtering and normalization, a fork of BROAD Institute's GTEx pipeline repository for calculating PEER factors, FastQTL for Cis-QTL analysis, SMR for SMR analysis, Hi-C Pro for analysis of Hi-C data, BLASTn for conservation analysis, DESeq2 for differential expression analysis, CEMITools for network analysis and module generation and Cytoscape for visualization.

For manuscripts utilizing custom algorithms or software that are central to the research but not yet described in published literature, software must be made available to editors/reviewers. We strongly encourage code deposition in a community repository (e.g. GitHub). See the Nature Research guidelines for submitting code & software for further information.

Data

Policy information about availability of data

All manuscripts must include a data availability statement. This statement should provide the following information, where applicable:

- Accession codes, unique identifiers, or web links for publicly available datasets
- A list of figures that have associated raw data
- A description of any restrictions on data availability

The data that support the findings of this study are available from the corresponding author upon reasonable request.

Field-specific reporting

Please select the one below that is the best fit for your research. If you are not sure, read the appropriate sections before making your selection.

- [x] Life sciences
- [ ] Behavioural & social sciences
- [ ] Ecological, evolutionary & environmental sciences

For a reference copy of the document with all sections, see nature.com/documents/nr-reporting-summary-flat.pdf

Life sciences study design

All studies must disclose on these points even when the disclosure is negative.

Sample size	According to the variability of metabolic parameters, group size was determined based on previous studies using similar assays within the laboratory and pilot experiments. The precise number of animals used are given in the figure legend.
Data exclusions	Animal data were excluded from experiments based on pre-established criteria of visible abnormal liver structure during sample harvest or other health issues such as fighting wounds or infections.
Replication	Replicate experiments were successful.
Randomization	We did not use randomization to assign animals to experimental groups.
Blinding	Experimenters were not blinded to treatment group.

Reporting for specific materials, systems and methods

We require information from authors about some types of materials, experimental systems and methods used in many studies. Here, indicate whether each material, system or method listed is relevant to your study. If you are not sure if a list item applies to your research, read the appropriate section before selecting a response.

Materials & experimental systems

n/a	Involved in the study	
[ ]	[x]	Antibodies
[ ]	[x]	Eukaryotic cell lines
[x]	[ ]	Palaeontology
[ ]	[x]	Animals and other organisms
[x]	[ ]	Human research participants
[x]	[ ]	Clinical data

Methods

n/a	Involved in the study	
[x]	[ ]	ChIP-seq
[x]	[ ]	Flow cytometry
[x]	[ ]	MRI-based neuroimaging

Antibodies

Antibodies used	HuR -RIP Validated Antibody (MilliporeSigma), RNA Pol II ChIP validated antibody (MilliporeSigma)
Validation	These antibodies have been validated by the providers.

Eukaryotic cell lines

Policy information about cell lines

Cell line source(s)	293A cells (Invitrogen)
Authentication	The 293A cells have been routinely used for adenoviruses preparation in our lab
Mycoplasma contamination	Negative
Commonly misidentified lines (See ICLAC register)	N/A

Animals and other organisms

Policy information about studies involving animals; ARRIVE guidelines recommended for reporting animal research

Laboratory animals	TK-NOG mice were obtained from Taconic Biosciences. To prepare humanized TK-NOG mice, the TK-NOG mice at 8-9 weeks old received an i.p. injection of GCV at a dose of 25 mg/kg. One week later, 50ul volume of 1×10E6 human primary hepatocytes suspended in HBSS solution were transplanted via intra-splenic injection. 8-12 weeks after transplantation, the serum human albumin in the mice were measured as an index of the extent of human hepatocytes replacement. Male C57BL/6 (B6) mice were purchased from Jackson Laboratory at 8 weeks of age, and housed 3-5 mice per cage with free access to water and normal chow diet (NIH-31), and animals were acclimatized to the housing for 10-14 days before experiments.
Wild animals	N/A
Field-collected samples	N/A
Ethics oversight	All animal experiments were performed in accordance and with approval from the NHLBI Animal Care and Use Committee or the Animal Care Committee of the Central Institute for Experimental Animals (CIEA).

Note that full information on the approval of the study protocol must also be provided in the manuscript.
