# Supplementary material for: In vivo functional analysis of non-conserved human lncRNAs associated with cardiometabolic traits
Source: Nat Commun. 2020 Jan 2;11:45. doi: 10.1038/s41467-019-13688-z (PMC6940387; doi:10.1038/s41467-019-13688-z)
Supplement: Supplementary file 3 — Description of Additional Supplementary Files [file 41467_2019_13688_MOESM3_ESM.docx]

Description of Additional Supplementary Files

File name: Supplementary Data 1

Description: List of GWAS meta-analyses and liver cardiometabolic trait-associated lnc-eGenes.

File name: Supplementary Data 2

Description: List of cardiometabolic trait-associated lnc-eGenes identified by integration of active enhancer epigenetic marker and spatial chromatin interaction information.

File name: Supplementary Data 3

Description: List of cardiometabolic trait-associated lnc-eGenes with liver enriched expression

File name: Supplementary Data 4

Description: Full list of cardiometabolic trait-associated lnc-eGenes in each module and KEGG pathway analysis overrepresented by protein-coding genes within each co-expression module.

File name: Supplementary Data 5

Description: List of cardiometabolic trait-associated lnc-eGenes regulated by fasting.

File name: Supplementary Data 6

Description: List of proteins identified by LINC01018 pulldown and Mass-spectrometry.

File name: Supplementary Data 7

Description: Sequences of primers used.
